# Parameters of dual-layer spectral detector CT for differentiating pathological types of colorectal adenocarcinoma

**DOI:** 10.1186/s13244-026-02298-1

**Published:** 2026-05-15

**Authors:** Sisi Huang, Dongping Jiang, Shuang Tang, Yuquan Zheng, Shuwen Zheng, Xiaomin Liu, Yuting Liao, Lili Feng, Shanshan Lian

**Affiliations:** 1 Department of Radiology, State Key Laboratory of Oncology in South China, Guangdong Provincial Clinical Research Center for Cancer, Sun Yat-sen University Cancer Center, Guangzhou, P. R. China; 2 Clinical & Technical Solutions, Philips Healthcare, Guangzhou, P. R. China

**Keywords:** Colorectal cancer, Computed tomography, Dual energy, Mucinous carcinoma

## Abstract

**Objectives:**

To investigate the diagnostic performance of quantitative parameters derived from Dual-layer spectral detector CT (DLCT) in distinguishing between mucinous adenocarcinoma (MC) and adenocarcinoma not otherwise specified (AC) in colorectal adenocarcinoma (CRAC).

**Materials and methods:**

Patients with pathologically confirmed CRAC who underwent preoperative DLCT scanning were enrolled in this retrospective study from May 2022 to September 2024. A propensity score-matching (PSM) approach was employed to balance the important patient characteristics between the MC group and the AC group. DLCT quantitative parameters were compared between the MC and AC groups, and a multifactorial binary logistic stepwise forward regression analysis was conducted to identify independent factors. Receiver operating characteristic (ROC) curves were utilized to evaluate diagnostic efficacy.

**Results:**

A total of 260 patients were enrolled in this study. Following PSM, 23 patients with MC and 23 patients with AC were included in the analysis. All DLCT parameters were significantly lower in patients with MC compared to those with AC (all *p* < 0.01). Electron density (OR = 0.007, 95% CI: 0.000–0.295) and normalized iodine concentration (OR = 0.000, 95% CI: 0.000–0.067) in the venous phase were identified as independent predictors associated with different histopathological types. The combination of these two spectral parameters demonstrated significantly superior diagnostic efficacy compared to conventional CT attenuation values (*p* < 0.05), achieving an area under the curve of 0.949, with a sensitivity of 87.0% and a specificity of 91.3%.

**Conclusions:**

Quantitative spectral parameters derived from DLCT can effectively differentiate MC from AC preoperatively, exhibiting excellent diagnostic efficacy.

**Critical relevance statement:**

DLCT is a reliable diagnostic tool to preoperatively differentiate pathological types of colorectal cancer, thereby facilitating personalized treatment strategies.

**Key Points:**

- This study investigated the value of dual-layer spectral CT (DLCT) in differentiating the pathological types of colorectal cancer.
- Quantitative spectral parameters demonstrated excellent accuracy in the diagnosis of mucinous adenocarcinoma.
- Preoperative DLCT can help clinicians in formulating personalized treatment strategies.

**Graphical Abstract:**

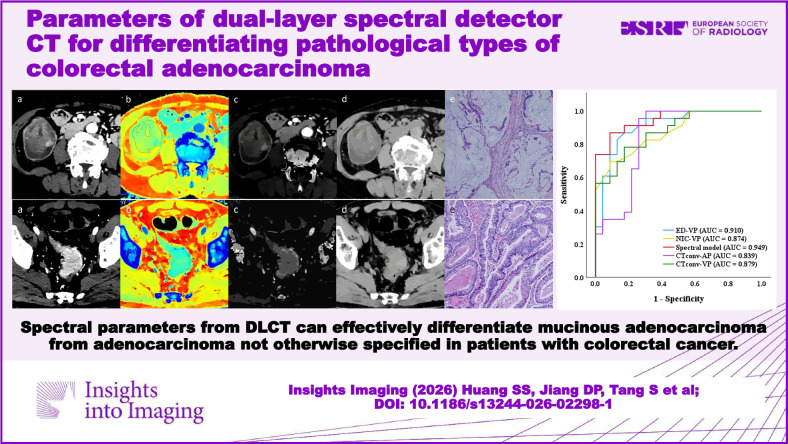

## Introduction

Colorectal cancer (CRC) ranks among the most prevalent cancers worldwide and is the second leading cause of cancer-related deaths [1]. Adenocarcinoma is the most common type of colorectal cancer, of which mucinous adenocarcinoma (MC) is a unique subtype characterized by the presence of large amounts of extracellular mucus in the tumor, accounting for more than 50% of the tumor volume [2]. MC makes up approximately 10–20% of colorectal cancers and is more common in women and younger patients. Compared to classic adenocarcinoma, MC is typically located in the proximal colon and is often diagnosed at an advanced stage [2]. In terms of treatment, MC shows poor response to traditional cytotoxic chemotherapy and radiotherapy, which may be related to its unique genetic mutations and copy number variations [3, 4]. Therefore, accurately identifying the pathologic subtypes is of importance to guide personalized treatment for patients with CRC.

Computed tomography (CT) and magnetic resonance imaging (MRI) are commonly used for the diagnosis and staging of colorectal cancer. Compared with the low accuracy of biopsy pathology [5, 6], multiparametric MRI has been reported to be more advantageous in detecting and evaluating MC. T2-weighted images can clearly show the characteristics of MC, which may be less apparent on conventional CT imaging [6]. Studies show that the T1 relaxation time and apparent diffusion coefficient (ADC) values of MC are significantly higher than those of non-mucinous adenocarcinoma in patients with rectal adenocarcinoma, providing clinicians with a valuable diagnostic tool [7]. However, MRI is routinely used in the evaluation of rectal cancer, but not in colon cancer.

Dual-layer spectral detector CT (DLCT) is an advanced imaging technique that leverages the energy dependence of X-ray attenuation to achieve superior material differentiation capabilities [8]. Compared with conventional CT, DLCT is capable of providing a greater amount of quantitative imaging information that is beneficial for diagnosis, including iodine concentration (IC), effective atomic number (Zeff), and electron density (ED), among others. Studies have demonstrated that the quantitative parameters of DLCT hold significant clinical application value in evaluating colorectal cancer, including T stage, differentiation grade, lymph node metastasis, and prognosis [9–12]. However, to the best of our knowledge, there are currently no published studies reporting the use of DLCT parameters for identifying pathological subtypes of colorectal adenocarcinoma (CRAC). Therefore, the objective of this study was to evaluate the diagnostic efficacy of preoperative DLCT parameters in distinguishing MC from adenocarcinoma not otherwise specified (AC), the latter being the most prevalent form of CRAC.

## Materials and methods

### Patients

Approval for this retrospective study was granted by the institutional review board (B2025-474-01), and the requirement for informed consent was waived.

**Fig. 1:**
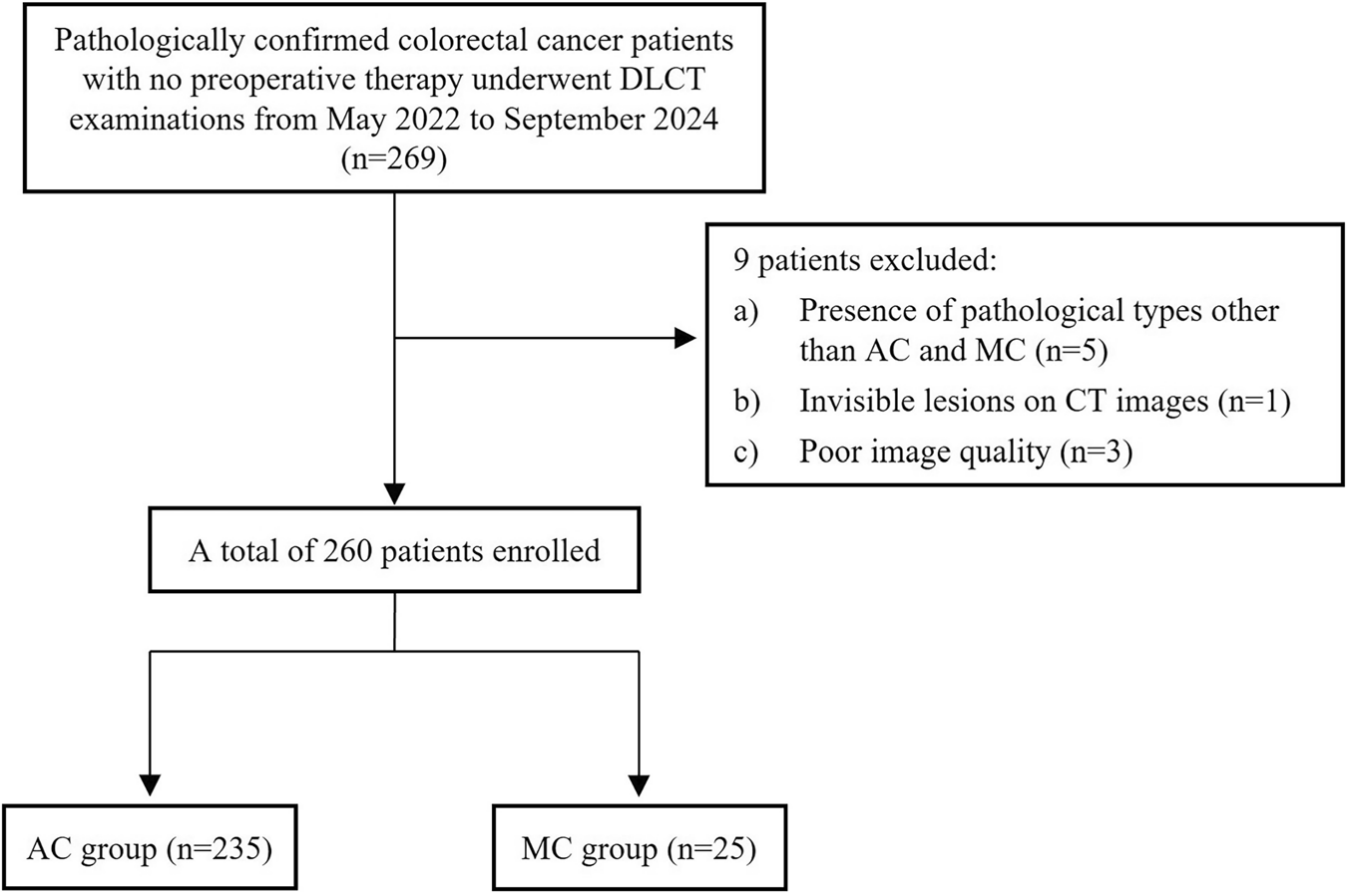
Flowchart of patient selection. DLCT, dual-layer spectral detector CT; MC, mucinous adenocarcinoma; AC, adenocarcinoma not otherwise specified

From May 2022 to September 2024, all consecutive patients who underwent preoperative DLCT examination and were pathologically diagnosed with CRAC in our institution were reviewed. The preliminary evaluation of all CT images was performed by a single radiologist who did not participate in any subsequent measurements. Inclusion criteria were: (1) patients who had surgical resection at our hospital without any preoperative antitumor treatment; (2) preoperative abdominal-pelvic DLCT examination was performed within 2 weeks before surgery; (3) complete clinical and pathological information was available. Exclusion criteria included: (1) presence of pathological types other than AC and MC; (2) lesions that were invisible on CT images; (3) poor image quality. Finally, a total of 260 patients were enrolled in this study. The flowchart of patient selection is shown in Fig. 1. Clinical and pathological information was obtained from the hospital medical record system.

### CT examination

CT scans were performed with a DLCT scanner (IQon Spectral CT, Philips Healthcare). The imaging parameters were specified as follows: a tube voltage of 120 kVp, a gantry rotation time of 0.5 s, and an effective tube current-time product ranging between 120 and 250 mAs. The pitch was set at 1.0, and the detector-row collimation was configured to 64 × 0.625 mm. For contrast-enhanced imaging, a nonionic contrast agent (Ultravist 370 mg/mL) was administered via the right ulnar vein at a rate of 2.3 mL/s, with a dosage of 1.35 mL/kg of body weight, followed by a 30 mL saline flush at the same flow rate. Following the administration of the contrast material, scans for the arterial phase (AP) and venous phase (VP) were initiated at 33 and 65 s, respectively.

After scanning, spectral-based images (SBIs) were used to reconstruct 40-keV and 100-keV virtual monochromatic images (VMIs), conventional CT (CT_conv_) images, IC maps, Zeff maps, ED maps, and virtual non-contrast (VNC) maps. The slice thickness and spacing were both 1 mm.

### Image analysis

**Fig. 2:**
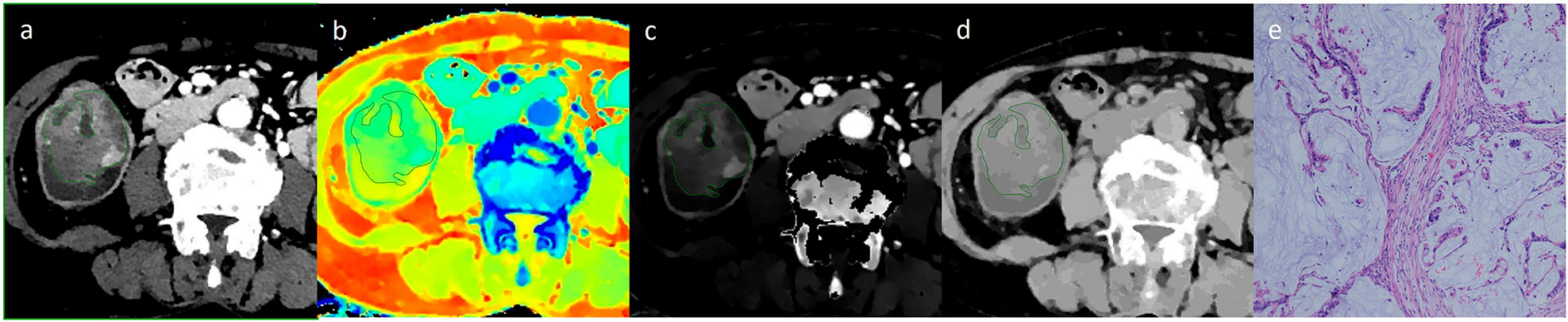
Dual-layer spectral detector CT images and pathological sections obtained in a 78-year-old female patient with colorectal mucinous adenocarcinoma. The ROI was delineated along the contour of the lesion in the 40-keV virtual monochromatic image (**a**), and copied to the effective atomic number image (**b**), iodine concentration image (**c**), and electron density image (**d**) in the venous phase. The histopathology (HE, magnification: × 100) demonstrates large mucin pools around the cancer cells (**e**)

**Fig. 3:**
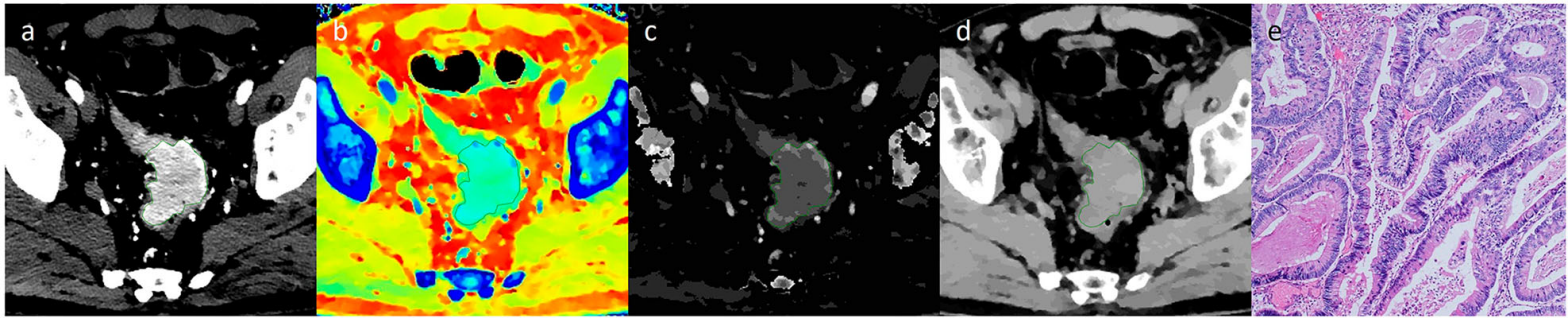
Dual-layer spectral detector CT images and pathological sections obtained in a 64-year-old male patient with colorectal adenocarcinoma not otherwise specified. The ROI was delineated along the contour of the lesion in the 40-keV virtual monochromatic image (**a**), and copied to the effective atomic number image (**b**), iodine concentration image (**c**), and electron density image (**d**) in the venous phase. The histopathology (HE, magnification: × 100) demonstrates moderately differentiated adenocarcinoma (**e**)

The CT images were independently analyzed by two radiologists with 5 and 10 years of experience, respectively, who were unaware of the pathological results. In the 40 keV VMIs, a freehand region of interest (ROI) was delineated along the periphery of the lesion at the slice exhibiting the largest axial diameter, with careful avoidance of intestinal contents, blood vessels, visible necrotic regions, and calcifications to the greatest extent possible. Another ROI was placed on the abdominal aorta or iliac artery at the same level as the lesion. The ROIs were then copied to the CT_conv_ images, 100-keV VMIs, IC maps, Zeff maps, ED maps, and VNC maps (Figs. 2, 3). IC refers to the iodine per unit volume in the ROI, and it was normalized (NIC) against that in the aorta or iliac artery according to the following formula: NIC = IC_lesion_ / IC_aorta_. Zeff is the atomic number of a hypothetical single element that would exhibit the same X-ray attenuation characteristics as the composite material. ED is the number of electrons per unit volume, expressed as a ratio relative to water. VNC is generated by mathematically removing the iodine signal from a contrast-enhanced scan to mimic a true non-contrast scan. The slope of the spectral Hounsfield unit curve (λ_HU_) was calculated as follows: λ_HU_ = (CT_40keV_ − CT_100keV_)/60. The average value of each parameter measured by the two radiologists was calculated as the final value.

### Statistical analysis

All statistical analyses were performed using SPSS software (version 26.0). The propensity score-matching (PSM) method was used to balance the important patient characteristics between groups. The propensity score was calculated based on covariates, including age, sex, gender, tumor location, carcinoembryonic antigen (CEA), pathological T (pT) stage and pathological N (pN) stage, using a multivariable logistic regression model. Patients with AC and MC were matched based on their propensity scores using nearest neighbor matching without replacement, with a caliper width of 0.03 and a matching ratio of 1:1. Continuous variables with normal distribution were presented as mean ± standard deviation and were compared by independent *t*-test between two groups or two-way analysis of variance among multiple groups, whereas those with non-normal distribution are presented as median and interquartile range and were compared by Mann–Whitney U test. Differences in categorical variables were analyzed by the chi-square test or Fisher’s exact test. Intraclass correlation coefficients (ICC) were used to assess the interobserver reliability of each DLCT parameter. A multifactorial binary logistic stepwise forward regression analysis was conducted on indicators that demonstrated statistical significance in univariate analysis to identify independent factors. The diagnostic performance was assessed using receiver operating characteristic (ROC) curve analysis, and areas under the curves (AUCs) were compared by DeLong’s test. Statistically significant differences were defined as *p* < 0.05.

## Results

### Patient characteristics

A total of 260 CRAC patients were enrolled in this study, with a mean age of 60 ± 12 years. Of these, 156 were male and 104 were female. Pathological diagnosis identified 25 patients with MC, representing a prevalence rate of 9.6%, while the remaining 235 patients were diagnosed with AC. Table 1 presents the clinical and pathological characteristics of both the AC and MC groups. The pT stage was significantly more advanced in the MC group compared to the AC group (*p* < 0.001).

**Table 1:** Clinical characteristics of patients before and after propensity score-matching

Characteristics	Before matching			After matching		
	AC (*n* = 235)	MC (*n* = 25)	*p*-value	AC (*n* = 23)	MC (*n* = 23)	*p*-value
Gender			0.668			0.767
Male	142 (60.4)	14 (56.0)		12 (52.2)	13 (56.5)	
Female	93 (39.6)	11 (44.0)		11 (47.8)	10 (43.5)	
Age (years, Mean ± SD)	60 ± 11	56 ± 17	0.199	59 ± 10	58 ± 16	0.750
CEA			0.679			0.359
Normal	150 (63.8)	17 (68.0)		13 (56.5)	16 (69.6)	
Abnormal	85 (36.2)	8 (32.0)		10 (43.5)	7 (30.4)	
pT stage			< 0.001			0.978
T1-2	47 (20.0)	3 (12.0)		2 (8.7)	3 (13.0)	
T3	174 (74.0)	15 (60.0)		17 (73.9)	15 (65.2)	
T4	14 (6.0)	7 (28.0)		4 (17.4)	5 (21.7)	
pN stage						
N0	146 (62.1)	13 (52.0)	0.323	11 (47.8)	13 (56.5)	0.555
Metastatic status						
M0	226 (96.2)	22 (88.0)	0.097	22 (95.7)	21 (91.3)	1.000
Tumor location			0.716			0.227
Colon	177 (75.3)	18 (72.0)		12 (52.2)	16 (69.6)	
Rectum	58 (24.7)	7 (28.0)		11 (47.8)	7 (30.4)	

*AC* adenocarcinoma not otherwise specified, *CEA* carcinoembryonic antigen, *MC* mucinous adenocarcinoma, *pT* pathological tumor stage, *pN* pathological nodal stage

### Interobserver agreement

The interobserver agreement for the DLCT parameters was excellent, with ICC ranging from 0.875 (95% CI: 0.786–0.929) to 0.981 (95% CI: 0.966–0.989). Details of the ICC values are provided in Table S1.

### Comparison of DLCT parameters between different pT stages

The CT_conv_-AP, ED-AP, NIC-AP, VNC-AP, CT_conv_-VP, Zeff-VP, ED-VP, IC-VP, NIC-VP, VNC-VP, and attenuation on 40-keV VMIs in VP were significantly different among pT1-2, pT3, and pT4 stages (all *p* < 0.05), and all of them were lower at more advanced pT stages. In patients with AC, CT_conv_-AP (*p* = 0.032), NIC-AP (*p* = 0.034), VNC-AP (*p* = 0.016), ED-VP (*p* = 0.030), and VNC-VP (*p* = 0.006) showed significant differences among pT1-2, pT3, and pT4 stages. In patients with MC, there was no significant difference in the DLCT parameters among different pT stages (all *p* > 0.05) (Table S2).

### Quantitative DLCT parameters between the AC and MC groups

Following PSM, 23 patients with MC and 23 patients with AC were included in the subsequent analysis, and patient characteristics, including gender, age, CEA, pT stage, pN stage, and tumor location, were balanced (*p* > 0.05) (Table 1).

The parameters of DLCT for patients with AC and MC are presented in Table 2. CT_conv_, Zeff, ED, IC, NIC, attenuation on 40-keV VMIs, λ_HU_, and VNC were significantly lower in patients with MC compared to those with AC, in both the arterial phase and venous phase (all *p* < 0.001).

**Table 2:** Spectral CT parameters between patients with AC and MC in the PSM cohort

Parameters	AC	MC	*p*-value
AP			
CT_conv_ (HU)	68.08 (65.52–91.30)	54.77 (50.52–66.24)	< 0.001
Zeff	7.96 (7.89–8.15)	7.80 (7.70–7.91)	< 0.001
ED	104.15 (103.96–104.37)	103.50 (103.22–103.83)	< 0.001
IC (mg/mL)	1.14 (1.01–1.51)	0.85 (0.69–1.04)	< 0.001
NIC	0.15 (0.13–0.19)	0.11 (0.09–0.14)	< 0.001
40 keV (HU)	136.51 (127.22–166.36)	103.81 (93.22–127.67)	< 0.001
λ_HU_	1.41 (1.26–1.88)	1.06 (0.86–1.33)	< 0.001
VNC	40.27 ± 3.55	32.86 ± 4.77	< 0.001
VP			
CT_conv_ (HU)	88.93 ± 12.62	69.66 ± 10.20	< 0.001
Zeff	8.32 ± 0.19	8.07 ± 0.15	< 0.001
ED	104.34 ± 0.35	103.59 ± 0.47	< 0.001
IC (mg/mL)	104.34 ± 0.33	103.63 ± 0.48	< 0.001
NIC	0.41 ± 0.08	0.29 ± 0.06	< 0.001
40 keV (HU)	202.38 ± 36.39	151.27 ± 27.51	< 0.001
λ_HU_	2.34 ± 0.51	1.71 ± 0.38	< 0.001
VNC	41.11 ± 3.26	33.92 ± 4.69	< 0.001

Data are presented as mean ± standard deviation for normally distributed data and median (25th percentile–75th percentile) for non-normally distributed data

*AC* adenocarcinoma not otherwise specified, *AP* arterial phase, *CT*_*conv*_ conventional CT, *ED* electron density, *IC* iodine concentration, *MC* mucinous adenocarcinoma, *NIC* normalized iodine concentration, *VP* venous phase, *VNC* virtual non-contrast, *Zeff* effective atomic number, *λHU* the slope of the spectral Hounsfield unit curve

Multivariate logistic analysis indicated that ED-VP (OR = 0.007, 95% CI: 0.000–0.295, *p* = 0.009) and NIC-VP (OR = 0.000, 95% CI: 0.000–0.067, *p* = 0.027) were independent predictors for distinguishing MC from AC, as detailed in Table 3. Based on the findings from the multivariate logistic regression analysis, a combined spectral model was subsequently developed.

**Table 3:** Multifactorial binary logistic stepwise forward regression analysis of spectral CT parameters for prediction of mucinous adenocarcinoma

Parameters	B	SE	Wald	*p*-value	OR	95% CI of OR
ED-VP	−4.92	1.887	6.795	0.009	0.007	0.000–0.295
NIC-VP	−23.449	10.584	1.908	0.027	0.000	0.000–0.067

*B* biased regression coefficient, *CI* confidence interval, *ED* electron density, *NIC* normalized iodine concentration, *OR* odds ratio, *SE* standard error, *VP* venous phase

### Diagnostic performance of DLCT parameters

**Fig. 4:**
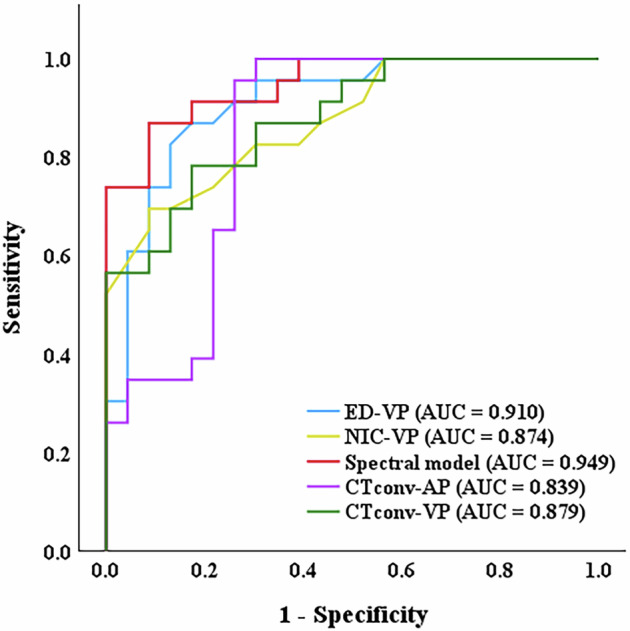
Receiver-operating characteristic curves of spectral CT parameters in predicting mucinous adenocarcinoma in the PSM cohort. AP, arterial phase; CT_conv_, conventional CT; ED, electron density; NIC, normalized iodine concentration; PSM, propensity score-matching; VP, venous phase

ROC curves for the independent predictors, the combined spectral model, CT_conv_-AP, and CT_conv_-VP are plotted in Fig. 4, while their diagnostic efficacy in differentiating MC from AC is shown in Table 4. The AUC for ED-VP in distinguishing MC from AC was 0.910 (95% CI: 0.826–0.995), with an optimal diagnostic threshold of 104.035, a sensitivity of 82.6%, and a specificity of 87.0%. The AUC for NIC-VP was 0.874 (95% CI: 0.777–0.995), with an optimal diagnostic threshold of 0.375, a sensitivity of 69.6%, and a specificity of 91.3%. The combined spectral model, which integrates ED-VP and NIC-VP, demonstrated a higher AUC of 0.949 (95% CI: 0.893–1.000), with a sensitivity of 87.0% and a specificity of 91.3%. DeLong’s test revealed that the AUC of the spectral model was significantly higher than those of CT_conv_-AP (0.949 vs. 0.839, *p* = 0.049) and CT_conv_-VP (0.949 vs. 0.879, *p* = 0.036).

**Table 4:** Diagnostic performance of spectral CT parameters for differentiating MC from AC

Parameters	AUC	95% CI	Cutoff value	Sensitivity	Specificity	Accuracy
ED-VP	0.910	0.826–0.995	104.035	0.826	0.870	0.826
NIC-VP	0.874	0.777–0.995	0.375	0.696	0.913	0.761
Spectral model	0.949	0.893–1.000	0.527	0.870	0.913	0.891
CT_conv_-AP	0.839	0.716–0.963	61.240	0.957	0.739	0.804
CT_conv_-VP	0.879	0.784–0.974	78.030	0.783	0.826	0.804

*AC* adenocarcinoma not otherwise specified, *AUC* area under the curve, *AP* arterial phase, *CI* confidence interval, *CT*_*conv*_ conventional CT, *ED* electron density, *MC* mucinous adenocarcinoma, *NIC* normalized iodine concentration, *VP* venous phase

## Discussion

In this study, we conducted a preliminary investigation into the diagnostic efficacy of DLCT parameters for determining the pathological type of CRAC. All quantitative parameters derived from DLCT, including CT_conv_, IC, NIC, Zeff, ED, λ_HU_, and VNC, exhibited significant differences between MC and AC. ED-VP and NIC-VP were independent predictors for distinguishing MC from AC, and the combination of these two demonstrated excellent diagnostic performance.

We observed that NIC-AP and ED-VP were significantly lower at more advanced pT stages in patients with AC. In contrast, Chen et al and Sun et al reported that colorectal adenocarcinoma at pT3 or pT4 stage demonstrated higher IC values than those at pT1-2 stages [13, 14]. Possible reasons for this contradiction might be different ROI placement methods and the presence of necrotic area in advanced pT stages, which was inevitable during ROI placement. Other DLCT parameters, including ED-AP, VNC-AP, Zeff-VP, IC-VP, NIC-VP, VNC-VP, and attenuation on 40-keV VMIs, were also significantly lower at more advanced pT stages in the whole cohort. This is probably due to the presence of MC, which demonstrated more advanced pT stages and lower values of DLCT parameters compared to AC. On the other hand, no significant difference was observed for the MC group, which may be due to the influence of varying mucus contents. Since there is a significant difference in pT stages between the AC and MC groups, PSM was applied to eliminate this bias.

MC is recognized as a distinct subtype of colorectal cancer, characterized by unique histological features and clinical behavior. In our study, it was observed that patients with MC were diagnosed at a significantly more advanced stage compared to those with AC, and the lesions were more frequently located in the colon than in the rectum, aligning with previous research findings [15, 16]. It has been reported that patients with MC exhibit a diminished response to palliative or adjuvant chemotherapy compared to those with AC [2]. Thus, precise diagnosis and specialized treatment are necessary for patients with MC. Ko et al [17] reported that specific CT features, such as more severe bowel-wall thickening, more areas with hypoattenuation, heterogeneous contrast enhancement and intratumoral calcification, were significantly associated with MC. However, these CT findings were qualitative and relied on subjective judgment, and the diagnostic accuracy was also limited. The findings of this study indicated that quantitative spectral parameters derived from DLCT exhibited excellent diagnostic performance in differentiating MC from AC, significantly outperforming conventional CT attenuation values, thereby offering a more reliable diagnostic approach for MC.

ED reflects the probability of electrons being present at specific locations and can provide information pertaining to tissue composition. It is typically employed for patient dose calculations during the planning of radiotherapy treatments [18]. In recent years, several studies have shown that ED plays a role in the diagnosis of metastatic lymph nodes and the assessment of tumor invasiveness [19–22]. Our results revealed that ED is significantly lower in MC than in AC. Previous studies showed a linear relationship between ED and mass density [23, 24]. MC is characterized by an abundant mucinous component within the tumor parenchyma, which may be associated with a lower ED. As for contrast enhancement, its effect on ED was not clear. Nagano et al reported that IC and ED were not significantly correlated [20]. In our study, both ED and NIC were independent predictors of MC. Therefore, ED may reflect a property of tissue that is distinct from contrast enhancement.

The material decomposition capability enables spectral CT to quantitatively measure the iodine concentration of tissues in enhanced images [25]. NIC is considered to be able to reduce the influence of individual circulatory variability on the iodine content within the tumor [26]. IC has been suggested to be positively correlated with microvascular density and effectively reflects neovascularization and microcirculation in a tumor [26, 27]. Marcon et al confirmed that IC is positively correlated with microvascular density and could differentiate well between papillary and clear cell renal cell carcinoma [28]. However, there have been few reports regarding the tumor perfusion of MC. Shahid et al compared the contrast-enhancement ratios for mucinous and non-mucinous colorectal carcinoma on MRI, and no difference was observed [29]. Another study demonstrated that, on CT scans, the solid portion of MC was enhanced less than that of non-mucinous colorectal carcinoma [17]. This discrepancy may be due to the different mechanisms of contrast enhancement in CT and MR and the small sample size. Our study found that both NIC in AP and VP are significantly lower in MC than in AC, suggesting a lower microvascular density in MC than in AC.

There are several limitations in our study. First, this is a single-center study, and the sample size is relatively small, especially the small number of patients with MC. It is necessary to validate our results in a larger sample. Second, ED and Zeff were derived from AP and VP images, which may be affected by contrast agents. In addition, this study did not investigate the diagnostic performance of conventional nonenhanced CT but rather VNC. The predictive value of ED and Zeff on non-contrast images and conventional nonenhanced CT needs to be further investigated. Third, due to the limitation of data, a 1:1 matching ratio was used instead of higher ratios, which resulted in a smaller overall sample size and may have led to the loss of some data information.

In conclusion, quantitative spectral parameters derived from DLCT showed excellent performance in preoperatively distinguishing MC from AC in patients with CRAC, and outperformed conventional CT attenuation values.

## Supplementary information

Supplementary information

## Data Availability

The data are available from the corresponding author upon reasonable request.

## Abbreviations

AC: Adenocarcinoma not otherwise specified
AP: Arterial phase
AUC: Area under the curve
CEA: Carcinoembryonic antigen
CRAC: Colorectal adenocarcinoma
CRC: Colorectal cancer
DLCT: Dual-layer spectral detector computed tomography
ED: Electron density
IC: Iodine concentration
ICC: Intraclass correlation coefficients
MC: Mucinous adenocarcinoma
NIC: Normalized iodine concentration
PSM: Propensity score-matching
ROC: Receiver operating characteristic
ROI: Region of interest
VMI: Virtual monochromatic image
VNC: Virtual non-contrast
VP: Venous phase
Zeff: Effective atomic number
