# Supplementary material for: Parameters of dual-layer spectral detector CT for differentiating pathological types of colorectal adenocarcinoma
Source: Insights Imaging. 2026 May 15;17:130. doi: 10.1186/s13244-026-02298-1 (PMC13179393; doi:10.1186/s13244-026-02298-1)
Supplement: Supplementary file 1 — Supplementary information [file 13244_2026_2298_MOESM1_ESM.pdf]

Parameters of dual-layer spectral detector CT for differentiating pathological types of colorectal adenocarcinoma

ELECTRONIC SUPPLEMENTARY MATERIAL

Table S1. Interobserver agreement for the spectral CT parameters

Parameters	ICC (95%CI)	*p*
AP		
CT_{conv}	0.967(0.942-0.982)	<0.001
Zeff	0.977(0.959-0.987)	<0.001
ED	0.927(0.872-0.959)	<0.001
IC	0.981(0.966-0.989)	<0.001
NIC	0.945(0.874-0.973)	<0.001
40keV	0.875(0.786-0.929)	<0.001
λ_{HU}	0.979(0.963-0.988)	<0.001
VNC	0.914(0.850-0.952)	<0.001
VP		
CT_{conv}	0.962(0.933-0.979)	<0.001
Zeff	0.977(0.958-0.987)	<0.001
ED	0.920(0.861-0.955)	<0.001
IC	0.978(0.961-0.988)	<0.001
NIC	0.964(0.935-0.980)	<0.001
40keV	0.975(0.956-0.986)	<0.001
λ_{HU}	0.976(0.956-0.986)	<0.001
VNC	0.908(0.840-0.948)	<0.001

AP arterial phase, CI confidence interval, CT_{conv} conventional CT, ED electron density, ICC intraclass correlation coefficient, IC iodine concentration, NIC normalized iodine concentration, VP venous phase, VNC virtual non-contrast, Zeff effective atomic number, λHU the slope of the spectral Hounsfield unit curve

Table S2. Spectral CT quantitative parameters between different pT stages

Parameters	Whole cohort				AC				MC			
	**T1-2 (n=50)**	**T3 (n=189)**	**T4 (n=21)**	***p***	**T1-2 (n=47)**	**T3 (n=174)**	**T4 (n=14)**	***p***	**T1-2 (n=3)**	**T3 (n=15)**	**T4 (n=7)**	***p***
AP												
CT_{conv} (HU)	74.39 ± 15.24	68.75 ± 11.78	63.81 ± 17.01	0.009	75.54 ± 14.99	69.69 ± 11.49	69.02 ± 15.81	0.032	56.43 ± 2.59	57.82 ± 9.63	53.38 ± 15.26	0.662
Zeff	8.03 ± 0.31	7.98 ± 0.20	7.93 ± 0.23	0.320	8.04 ± 0.31	7.99 ± 0.20	8.00 ± 0.22	0.509	7.80 ± 0.07	7.84 ± 0.16	7.77 ± 0.17	0.619
ED	104.10 ± 0.55	103.95 ± 0.46	103.66 ± 0.69	0.039	104.14 ± 0.53	103.99 ± 0.43	103.84 ± 0.58	0.051	103.39 ± 0.45	103.48 ± 0.45	103.30 ± 0.81	0.800
IC (mg/mL)	1.31 ± 0.60	1.19 ± 0.38	1.11 ± 0.44	0.266	1.34 ± 0.61	1.21 ± 0.38	1.25 ± 0.44	0.379	0.85 ± 0.13	0.93 ± 0.28	0.81 ± 0.28	0.621
NIC	0.18 ± 0.08	0.15 ± 0.05	0.14 ± 0.05	0.017	0.19 ± 0.09	0.15 ± 0.05	0.15 ± 0.05	0.034	0.11 ± 0.01	0.12 ± 0.06	0.10 ± 0.04	0.608
40keV (HU)	151.76 ± 50.92	139.56 ± 34.30	129.82 ± 42.66	0.160	154.71 ± 51.11	141.88 ± 34.01	144.26 ± 41.02	0.288	105.49 ± 6.16	112.60 ± 25.68	100.94 ± 30.99	0.616
λ_{HU}	1.63 ± 0.74	1.48 ± 0.48	1.38 ± 0.55	0.230	1.66 ± 0.75	1.51 ± 0.47	1.56 ± 0.54	0.485	1.08 ± 0.13	1.16 ± 0.35	1.01 ± 0.36	0.633
VNC	39.66 ± 5.32	37.95 ± 4.45	35.03 ± 6.45	0.005	40.17 ± 5.02	38.37 ± 4.21	36.83 ± 5.30	0.016	31.70 ± 3.51	33.12 ± 4.44	31.44 ± 7.42	0.826
VP												
CT_{conv} (HU)	88.60 ± 11.33	86.30 ± 11.18	77.42 ± 18.05	0.015	89.79 ± 10.62	87.66 ± 10.29	83.45 ± 14.53	0.104	70.06 ± 1.85	70.56 ± 9.11	65.36 ± 19.35	0.853
Zeff	8.32 ± 0.17	8.30 ± 0.17	8.20 ± 0.24	0.021	8.33 ± 0.17	8.32 ± 0.16	8.28 ± 0.17	0.600	8.08 ± 0.09	8.08 ± 0.14	8.02 ± 0.27	0.759
ED	104.28 ± 0.43	104.14 ± 0.43	103.78 ± 0.74	0.011	104.33 ± 0.38	104.18 ± 0.40	103.99 ± 0.62	0.030	103.41 ± 0.22	103.66 ± 0.38	103.34 ± 0.82	0.395
IC (mg/mL)	1.88 ± 0.38	1.86 ± 0.37	1.64 ± 0.48	0.037	1.92 ± 0.37	1.90 ± 0.35	1.81 ± 0.37	0.630	1.36 ± 0.18	1.39 ± 0.28	1.29 ± 0.51	0.840
NIC	0.41 ± 0.09	0.40 ± 0.08	0.35 ± 0.11	0.022	0.42 ± 0.09	0.41 ± 0.08	0.40 ± 0.09	0.490	0.30 ± 0.03	0.30 ± 0.07	0.26 ± 0.08	0.353
40keV (HU)	201.76 ± 33.46	197.68 ± 32.69	176.04 ± 46.69	0.012	205.08 ± 31.61	201.45 ± 30.46	192.67 ± 36.34	0.418	149.70 ± 10.86	153.93 ± 25.68	142.77 ± 49.66	0.822
λ_{HU}	2.34 ± 0.47	2.30 ± 0.46	2.04 ± 0.60	0.096	2.38 ± 0.46	2.36 ± 0.43	2.25 ± 0.46	0.413	1.72 ± 0.18	1.73 ± 0.35	1.62 ± 0.66	0.695
VNC	40.80 ± 4.18	39.04 ± 4.22	35.61 ± 7.02	0.002	41.37 ± 3.58	39.44 ± 4.06	37.68 ± 5.81	0.006	31.95 ± 2.71	35.66 ± 3.89	31.49 ± 7.84	0.504

AC adenocarcinoma not otherwise specified, AP arterial phase, CT_{conv} conventional CT, ED electron density, IC iodine concentration, MC mucinous adenocarcinoma, NIC normalized iodine concentration, pT pathological tumor stage, VP venous phase, VNC virtual non-contrast, Zeff effective atomic number, λ_{HU} the slope of the spectral Hounsfield unit curve
